# Neutrophils as key regulators of tumor immunity that restrict immune checkpoint blockade in liver cancer

**DOI:** 10.20892/j.issn.2095-3941.2023.0019

**Published:** 2023-05-13

**Authors:** Mei Feng, Fangyanni Wang, Xinyu Liu, Tingting Hao, Ning Zhang, Mi Deng, Yisheng Pan, Ruirui Kong

**Affiliations:** 1 Translational Cancer Research Center, Peking University First Hospital, Beijing 100034, China; 2 Division of General Surgery, Peking University First Hospital, Beijing 100034, China; 3 International Cancer Institute, Peking University Health Science Center, Beijing 100191, China; 4 Yunnan Baiyao Group, Kunming 650500, China

**Keywords:** Liver cancer, neutrophil, PD-1, CD8^+^ T cell, exhaustion

## Abstract

**Objective:**

Liver cancer is a deadly malignancy associated with high mortality and morbidity. Less than 20% of patients with advanced liver cancer respond to a single anti-PD-1 treatment. The high heterogeneity of neutrophils in the tumor immune microenvironment in liver cancer may contribute to resistance to immune checkpoint blockade (ICB). However, the underlying mechanism remains largely unknown.

**Methods:**

We established an orthotopic liver cancer model by using transposable elements to integrate the oncogenes *Myc* and *Kras*^G12D^ into the genome in liver cells from conditional Trp53 null/null mice (pTMK/Trp53^−/−^). Flow cytometry and immunohistochemistry were used to assess the changes in immune cells in the tumor microenvironment. An *ex vivo* coculture assay was performed to test the inhibitory effects of tumor-associated neutrophils (TANs) on CD8^+^ T cells. The roles of neutrophils, T cells, and NK cells were validated through antibody-mediated depletion. The efficacy of the combination of neutrophil depletion and ICB was evaluated.

**Results:**

Orthotropic pTMK/Trp53^−/−^ mouse liver tumors displayed a moderate response to anti-Ly6G treatment but not PD-1 blockade. Depletion of neutrophils increased the infiltration of CD8^+^ T cells and decreased the number of exhausted T cells in the tumor microenvironment. Furthermore, depletion of either CD8^+^ T or NK cells abrogated the antitumor efficacy of anti-Ly6G treatment. Moreover, the combination of anti-Ly6G with anti-PD-L1 enhanced the infiltration of cytotoxic CD8^+^ T cells and thereafter resulted in a significantly greater decrease in tumor burden.

**Conclusions:**

Our data suggest that TANs may contribute to the resistance of liver cancer to ICB, and combining TAN depletion with T cell immunotherapy synergistically increases antitumor efficacy.

## Introduction

Liver cancer is the third leading cause of cancer-associated deaths worldwide, and is associated with poor prognosis and high recurrence rates^1–3^. It can be categorized into 3 major histological subtypes: hepatocellular carcinoma (HCC), intrahepatic cholangiocarcinoma (ICC), and combined HCC-ICC (CHC)^4^. Among these, HCC represents approximately 80%^5,6^, ICC represents 15%^7,8^, and CHC represents fewer than 5% of all cases of primary liver cancer^9^. Most patients with HCC are diagnosed in advanced or unresectable disease stages, and have limited treatment options^10^.

Immune checkpoint blockade (ICB) and therapies combined with ICB have been approved by the United States Food and Drug Administration (FDA) as a second-line treatment for HCC^11^. However, less than 20% of patients with HCC respond to ICB, and even fewer patients with ICC and CHC respond to anti-PD-1/PD-L1 immunotherapy^12^. Thus, the mechanism through which liver tumors resist ICB must urgently be identified.

Neutrophils play critical roles in many physiological and pathologic processes, including inflammation and cancer^13–20^. During infection, neutrophils rapidly traffic to inflammatory tissues, engulf pathogenic agents through phagocytosis, and heal tissue by releasing NETs and proteases^21–23^. Recently, after studying the heterogeneity of the liver tumor immune microenvironment (TIME), we defined 5 distinct subtypes of the TIME with different effects on the immune system: immune activation, myeloid suppression, stromal suppression, immune exclusion, and immune residence^24^. The myeloid suppression TIME of human liver cancer is highly enriched in neutrophils^25–29^. In human HCC, normal neutrophils can be induced by tumor cells to transform into tumor-associated neutrophils (TANs), which recruit immunosuppressive macrophages and Treg cells, and accelerate CD8^+^ T cell exhaustion^24,30^.

Our previous study has indicated that 3 subtypes of liver cancer frequently show loss of the *Tp53* gene, whereas genomic amplification of the oncogene *Myc* occurs in more than 70% of liver cancer cases^9^. In addition, Ctnnb1 activation has been found in multiple patients with HCC, whereas *Kras* mutations are present primarily in ICC^6,9^. In our previous study, we demonstrated that HCC can be induced by a combination of *Myc* and Ctnnb1 activation, and Trp53 knockout^24^.

Here, we established an orthotopic liver cancer model to mimic clinical HCC, ICC, and CHC subtypes with overexpression of *Myc* and *Kras*^G12D^, and deficiency in Trp53. Tumor-infiltrating neutrophils were found to suppress antitumor T cells in this mouse tumor model. The combination of anti-Ly6G with anti-PD-L1 treatment significantly decreased tumor burden. On the basis of our findings in this novel mouse liver cancer model, we propose a synergistic strategy for treating liver cancer by combining neutrophil depletion with ICB.

## Materials and methods

### Mice

All mouse experiments were approved by the Animal Care and Use Committee at Peking University First Hospital. All mice were maintained under pathogen-free conditions at an ambient temperature of 20–26 °C and a humidity of 30%–70% with a 12:12 h light-dark cycle, according to the guidelines of the animal facility at Peking University First Hospital. All mice were tested before the experiment to ensure that they were healthy and acclimated to the laboratory environment.

Trp53^fl/fl^ and Alb-Cre mice with a C57BL/6 background were purchased from Jackson Laboratory. Trp53^fl/fl^ mice were crossed with Alb-Cre mice to generate liver conditional Trp53 knockout (Trp53 cKO) mice. Sleeping beauty transposase (SB100) and transposon pT3-Neo-EF1a-GFP plasmids were purchased from Addgene. cDNA of the mouse *Myc* gene was cloned into the transposon vector through the MluI and SpeI restriction enzyme sites, thus producing the pT3-Neo-EF1a-*Myc* plasmid. Next, *Kras*^G12D^ fragments were obtained by PCR cloning of mouse cDNA. Subsequently, the *Myc* and *Kras*^G12D^ transposon plasmid (pT3-*Myc*-*Kras*^G12D^, pTMK) was generated *via* the AscI and NotI restriction sites. For pTMK-Luci plasmid construction, we linked the luciferase reporter gene to *Myc* *via* a T2A linker.

Plasmids for hydrodynamic tail vein (HDTV) injection were prepared with an EndoFree-Maxi Kit (Qiagen). For HDTV injection, a 30 μg DNA mixture (5:1 ratio of transposon to transposase-encoding plasmid) was suspended in 0.9% saline solution at a final volume equal to 10% of the body weight of the mice and was then injected into 8-week-old male Trp53 cKO mice *via* the tail vein within 5–7 s.

During the development of the pTMK/Trp53^−/−^ mouse models, the mouse body weights slightly decreased over the first 9 days after the plasmid injection, returned to their initial levels by 10 days, and then gradually increased as tumors grew. All pTMK/Trp53^−/−^ mice died from their liver tumors within 50 days. After anesthetization, palpable liver tumor nodules were detected in all mice. The number of liver tumor nodules was quantified, and the ratio of the liver weight to the body weight was calculated to assess the tumor mass.

### Depletion of immune cells

For anti-Ly6G therapy of pTMK/Trp53^−/−^ tumors, anti-Ly6G antibody (clone 1A8, BioXCell) or IgG2a isotype control (clone 2A3, BioXCell) at a dose of 12.5 μg per 100 μL 0.9% NaCl solution was administered daily *via* intraperitoneal injection, starting 7 days before HDTV injection of the pTMK plasmids. Thirty-two days later, the mice were sacrificed through carbon dioxide asphyxiation, and liver tumors were collected. The number of liver tumor nodules was calculated, and the ratio of liver weight to body weight was quantified.

For therapeutic treatment with anti-Ly6G blockade, mice were injected with pTMK-Luci plasmids to examine the bioluminescence signals, representing malignant tumor cells. At day 7, tumor-bearing mice were randomly divided into 2 groups receiving anti-Ly6G antibody or IgG2a isotype control treatment. For the anti-PD-1 monoclonal antibody experiment, mice were intraperitoneally administered anti-PD-1 (200 μg, clone RMP1-14, BioXCell) or IgG2b isotype control (200 μg, clone LTF-2, BioXCell) twice per week. For CD8^+^ T and natural killer (NK) cell depletion, anti-CD8a (200 μg, clone 2.43, BioXCell) or anti-NK1.1 (200 μg, clone PK136, BioXCell) was administered intraperitoneally into mice injected with pTMK-Luci plasmids.

Thirty-six days later, the mice were photographed with an *in vivo* imaging system (IVIS) to examine the bioluminescence signals, then sacrificed through carbon dioxide asphyxiation. Liver tumors were collected from the mice. For the combination of anti-PD-L1 and anti-Ly6G antibodies, anti-PD-L1 (200 μg, clone 10F.9G2, BioXCell) was used to treat the mice twice per week, whereas anti-Ly6G was used as described previously^12^. Thirty-two days later, the mice were sacrificed through carbon dioxide asphyxiation, and the liver tumors were collected from the mice.

### Bioluminescence imaging

For the therapeutic experiments, the lesions were detected through bioluminescence imaging with an IVIS spectrum system. After anesthetization with O_2_ and isoflurane, the mice received a 300 μL intraperitoneal injection of fresh D-luciferin (150 mg/kg; LABLEAD). After 5 min, luminescence was detected by an IVIS Lumina Series III (Caliper LifeSciences) with an exposure time of 60 s. The luciferase signal was quantified in Living Image software (Caliper LifeSciences). The bioluminescence signals from the tumors in the live mice were analyzed to estimate tumor mass.

### Isolation of neutrophils

Fresh mouse tumor and adjacent liver tissues from pTMK/Trp53^−/−^ mice were cut into approximately 1 mm^3^ pieces in RPMI 1640 medium (Thermo Fisher) with 10% fetal bovine serum (FBS, Gibco) and enzymatically digested with a MACS tumor dissociation kit (Miltenyi Biotec) for 30 min on a rotor at 37 °C, in accordance with the manufacturer’s instructions. After filtration through a 70 μm CellStrainer (BD) in RPMI 1640 medium, the suspended cells were centrifuged at 400 × *g* for 5 min. After removal of the supernatant, the cell pellets were resuspended in 36% Percoll (Sigma) solution (PBS supplemented with 2% FBS), then centrifuged at 500 × *g* for 15 min without braking. Subsequently, the immune cells were resuspended in Hanks’ balanced salt solution plus 2% FBS, and incubated with anti-Ly6G-PE antibodies at a ratio of 1 μg Abs to 1 × 10^6^ cells, then purified with magnetic beads. The purified TANs and adjacent liver neutrophils (ALNs) were incubated in RPMI 1640 medium plus 2% FBS for subsequent experiments.

Peripheral blood from pTMK/Trp53^−/−^ mice was diluted with Hanks’ balanced salt solution and added to Lymphoprep solution (STEMCELL). The cells were collected from the blood by centrifugation for 30 min at 400 × *g*. The red blood cells were lysed by resuspension in BRC lysis buffer (LABLEAD) for 15 min at 37 °C. The lysis was stopped with RPMI 1640 medium plus 2% FBS, and lysates were centrifuged at 400 × *g* for 10 min.

Spleens were mechanically disrupted, suspended in 1% FBS in PBS, and processed after lysis of the red blood cells. The cells were washed twice with PBS and filtered through a 70 μm CellStrainer (BD). The neutrophils were suspended at a concentration of 1 × 10^6^ cells per 100 μL RPMI 1640 medium plus 2% FBS, and incubated for 4 days. The viability and purity of the Ly6G^+^ neutrophils were determined to be > 90% through FACS analysis. Survival of neutrophils was quantified with a Cell Counting Kit-8 (Bestbio). The purified peripheral blood neutrophils (PBNs) and spleen neutrophils (SNs) were incubated in RPMI 1640 medium plus 2% FBS for subsequent experiments.

### Coculture of neutrophils and T cells

Mouse peripheral blood mononuclear cells from pTMK/Trp53^−/−^ peripheral blood were isolated by density gradient centrifugation through Ficoll-Paque solution. CD8^+^ T cells from peripheral blood mononuclear cells were purified with anti-CD8 magnetic beads (STEMCELL) and stimulated with 25 μg/mL CD3/CD28 T cell Activator (Life Technologies) and 50 U/mL rhIL-2 (STEMCELL) for 3–5 days. After 2 × 10^5^ CD8^+^ T cells were incubated with 5 × 10^5^ TANs, ALNs, or PBNs for 24 h, a mixed single-cell suspension was analyzed through flow cytometry.

### Flow cytometry

After being washed twice with PBS, 1 million cells were incubated with mouse FcR blocking reagents (BD), then incubated with fluorescently labeled antibodies on ice for 30 min. Samples were immediately analyzed with a FACSCanto flow cytometer (BD) in the flow cytometry core facility at Tuebingen. Doublets were excluded with height *vs.* area dot plots, and viable cells were additionally gated through Zombie UV exclusion. Ten thousand CD8^+^ T cells or CD45^+^ CD11b^+^ cells were further gated and collected to analyze the percentages of subpopulations. Data analysis was performed in FlowJo software.

### Immunohistochemistry (IHC)

Formalin-fixed and paraffin-embedded tissues sectioned to 4 μm were used for histological evaluation of liver tumors in a mouse model. Hematoxylin and eosin (H&E) staining was performed for each sample. For IHC, tissue slides were deparaffinized with xylene and rehydrated through a graded series of ethanol solutions (100%, 95%, and 70%). Subsequently, the slides were subjected to antigen retrieval by microwaving in a citric acid solution for 15 min. The primary antibodies anti-hepatocyte (Abcam, Cat#ab75677), anti-Epcam (Abcam, Cat#ab213500), anti-CD3 (Abcam, Cat#ab16669), anti-Ly6G (Servicebio, Cat#GB11229), anti-CD8 (Abcam, Cat#ab217344), anti-F4/80 (CST, Cat#70076), anti-Asma (Servicebio, Cat#BM0002), and anti-CD31 (Servicebio, Cat#GB113151) were used. Subsequently, the slides were incubated with secondary antibodies (1:1, 100 μL for each slide; HRP-anti-rabbit IgG, ZSGB, Cat#PV-6001, or HRP-anti-mouse IgG, ZSGB, Cat#PV-6002) for 10 min at room temperature. Multispectral images were scanned with a ZEISS AXIOSCAN 7 instrument. Cells of interest were quantified in Halo v3.4 (Indica Labs) or QuPath v0.2.0. Each section was evaluated by 2 or 3 experienced pathologists.

### Liver function tests

Serum alanine aminotransferase (ALT) and aspartate aminotransferase (AST) levels were determined with mouse ELISA kits (DingSheng) according to the manufacturer’s instructions, and the concentrations were calculated according to standard curves.

### Ethics approval

The animal study was performed in compliance with the guidance provided by the Animal Care Committee of Peking University First Hospital (Approval No. #J202105).

### Statistical analysis

All statistical calculations were performed in GraphPad Prism 7 software. Two-tailed Student’s *t-*tests and one-way ANOVA were used to determine the statistical significance of differences between 2 groups and among more than 2 groups, respectively. Mouse survival curves were generated with the Kaplan-Meier method, and the differences between groups were calculated with log-rank tests. Statistical significance was defined as *P* < 0.05. Error bars represent the standard error of the mean.

## Results

### *Kras* and *Myc* cooperatively induce orthotopic liver tumors in Trp53-deficient mice

**Figure 1:**
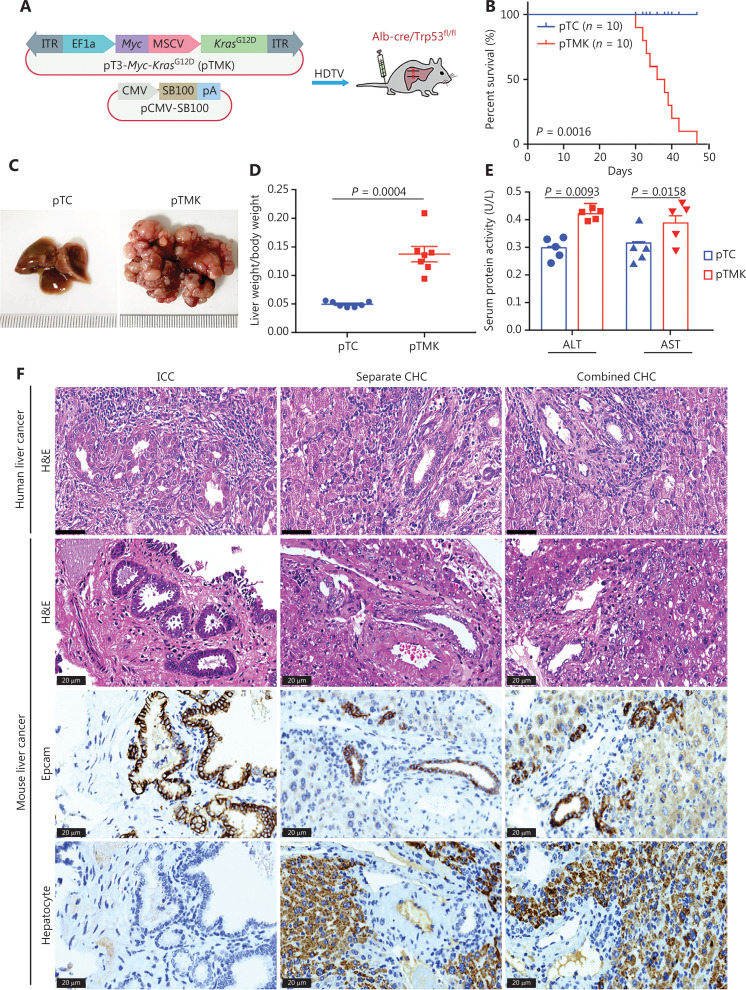
Cooperation between *Kras* and *Myc* in conditional Trp53 KO mice induces liver tumors. (A) Schematic of the mouse liver cancer model. The transposable vectors pTMK (encoding *Myc* and *Kras*^G12D^) plus the SB100 vector for expression of transposase were delivered into Alb-Cre × Trp53^fl/fl^ mice *via* HDTVi. (B) Survival graph of the pTC (control) and pTMK/Trp53^−/−^ mice. The number of mice per group is shown. Log-rank test was used to calculate *P*-values. (C) Representative macroscopic liver and tumor tissues from pTC and pTMK/Trp53^−/−^ mice. Rulers in the photo show a minimum unit of mm. (D) Quantification of tumor burden on the basis of the ratio of liver weight to body weight. (E) Detection of serum ALT and AST activity in pTC and pTMK/Trp53^−/−^ mice. (F) Representative H&E and IHC staining of HCC (hepatocyte) and ICC (Epcam) markers from human and mouse liver cancer tissues. Scale bar: 20 μm.

As we have previously reported^9^, the *Kras*, *Myc*, and *Tp53* genes have the highest frequency of genetic alterations, such as mutations, amplifications, or deletions, in human liver cancer, thus suggesting that these alterations may cooperatively drive hepatic tumorigenesis. We hypothesized that genetic alterations of these 3 genes might drive mouse liver tumorigenesis. First, we generated a transposon vector for coexpression of mouse *Myc* and mouse *Kras*^G12D^ (pTMK), in which *Myc* transcription was E2F-dependent, and *Kras*^G12D^ transcription was driven by an MSCV promoter (**Figure 1A**). Transposon-based pTMK together with a vector expressing SB100 were injected *via* the HDTV into the livers of conditional Trp53 knockout (AlbuminCre; Trp53^fl/fl^) mice (**Figure 1A**).

As expected, overexpression of *Myc* and *Kras*^G12D^ resulted in the development of liver cancer, and the mice had a median overall survival of 43 days (**Figure 1B**). Liver tumors were palpable in all mice after pTMK injection, whereas no nodules were detectable in the mice injected with the control vector over the course of 6 months (**Figure 1C**). The ratio of liver weight to body weight of the pTMK/Trp53^−/−^ mice was significantly higher than that of the control mice (**Figure 1D**). Moreover, the levels of ALT and AST in pTMK/Trp53^−/−^ mice were significantly higher than those in control mice (**Figure 1E**), thus suggesting that their liver function was impaired during tumor induction.

Furthermore, these tumors were characterized primarily as both ICC and CHC by histopathology (**Figure 1F**). Their ICC character was also established by strong staining for Epcam, thus indicating enrichment in biliary cells but a lack of hepatocytes. We compared the phenotypes of our mouse liver tumors with those of human liver tumors in terms of histopathology. The mouse ICC tumors were primarily well and moderately differentiated, with minimal gland formation and large pleomorphic cells—features similar to those of human ICC. The mouse CHC tumor tissues had a high density of tumor cells in a trabecular pattern, combined with ductular and papillary tumor structures—features similar to those of human combined or individual CHC. These data suggested that the pTMK/Trp53^−/−^ mouse tumors were similar to human ICC and CHC (**Figure 1F**). Together, these results indicated that *Kras*^G12D^ and *Myc* cooperatively induce a novel aggressive liver tumor with ICC and CHC phenotypes in Trp53^−/−^ mice.

### pTMK/Trp53^−/−^ liver cancer does not respond to ICB therapy

**Figure 2:**
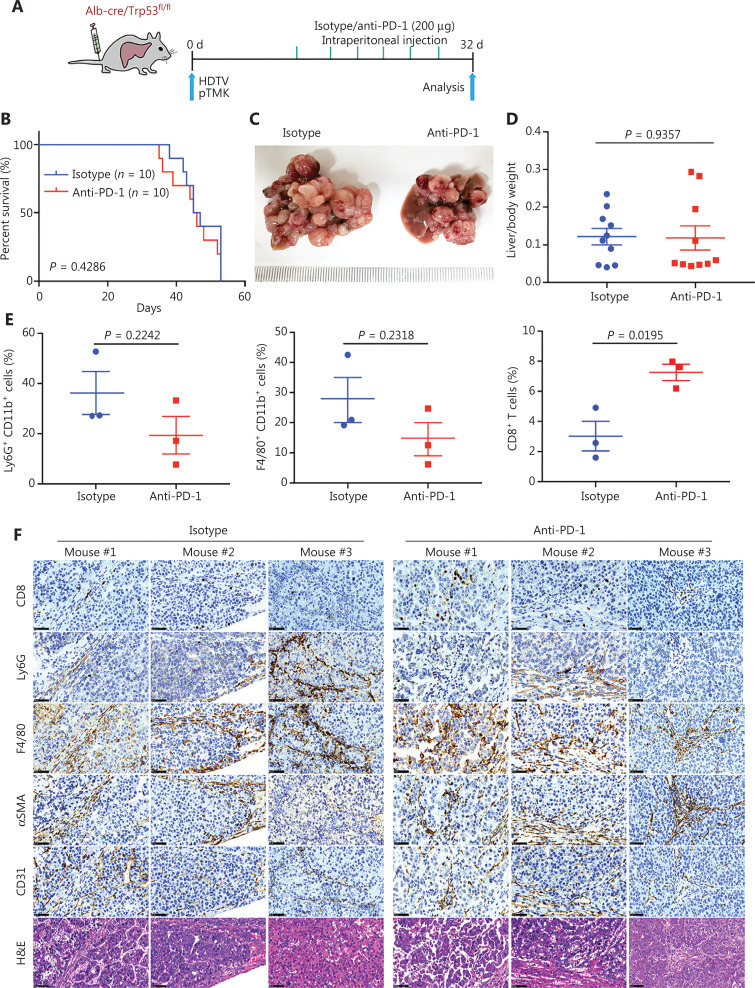
Driver gene-induced mouse liver cancer has a poor response to anti-PD-1 monotherapy. (A) Schematic of the experimental workflow. (B) Survival graph of pTMK/Trp53^−/−^ mice treated with isotype or anti-PD-1 monoclonal antibodies. The number of mice per group is shown. Log-rank test was used to calculate *P*-values. (C) Representative photographs of pTMK/Trp53^−/−^ mice treated with isotype or anti-PD-1 antibodies, showing microscopic tumors. Rulers in the photo show a minimum unit of mm. (D) The ratio of liver weight to body weight was calculated to assess tumor burden. (E) FACS analysis of populations of neutrophils, macrophages, and CD8^+^ T cells in the isotype and anti-PD-1 groups. (F) H&E and IHC of CD8, Ly6G, F4/80, αSMA, and CD31 in tumors or margin regions in mice treated with isotype control or anti-PD-1 antibodies. Scale bar: 20 μm.

Previous studies have shown that genetic alterations in liver cancer are associated with immune escape and resistance to immunotherapies^31–33^. We examined the therapeutic efficacy of ICB for pTMK/Trp53^−/−^ tumors (**Figure 2A and Supplementary Figure S1**). Similar to the clinical observations in patients with ICC and CHC, anti-PD-1 treatment did not suppress tumor growth or prolong survival or improve survival (**Figure 2B–2D**).

To discover the mechanism of resistance to ICB, we investigated the TIME of pTMK/Trp53^−/−^ liver tumors. Interestingly, CD8^+^ T cells slightly increased in response to anti-PD-1 treatment (**Figure 2E and Supplementary Figure S2**). Although other immune cells, such as CD11b^+^ myeloid cells, F4/80^+^ macrophages, Ly6G^+^ neutrophils, αSMA^+^ fibroblasts, and CD31^+^ blood vessel endothelial cells, did not change in response to anti-PD-1 treatment (**Figure 2E and 2F**), we observed enrichment with large amounts of neutrophils, macrophages, and fibroblasts in the tumor margin in the 2 groups (**Figure 2F**). Similarly, CD8^+^ T cells were colocalized in stromal regions of the tumors, thus suggesting that these cells might have been trapped or excluded by the myeloid cells and fibroblasts, and thus failed to exert tumor-killing functions (**Figure 2F**). Together, our findings indicated that this pTMK/Trp53^−/−^ orthotopic mouse liver cancer is similar to ICC/CHC tumors in humans and is resistant to ICB therapy.

### TANs promote CD8^+^ T cell exhaustion, thus facilitating mouse liver cancer growth

**Figure 3:**
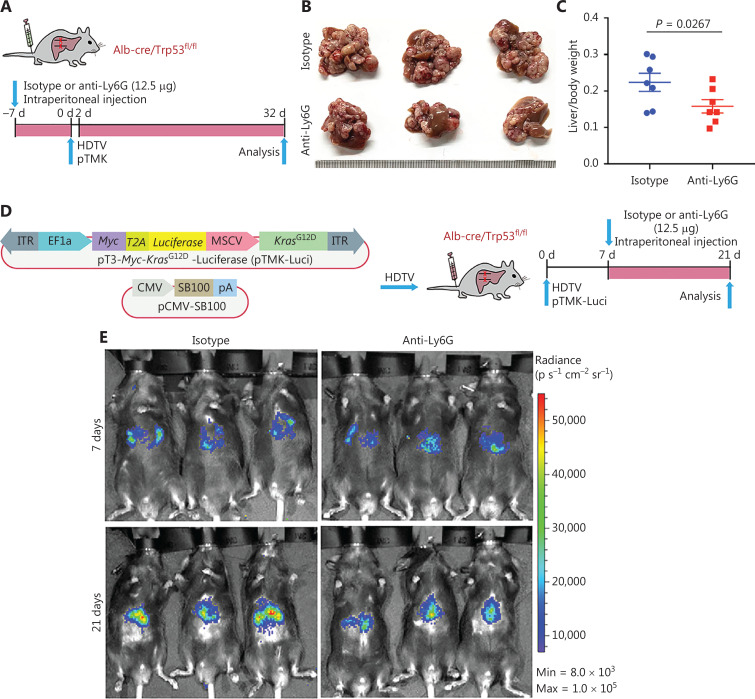

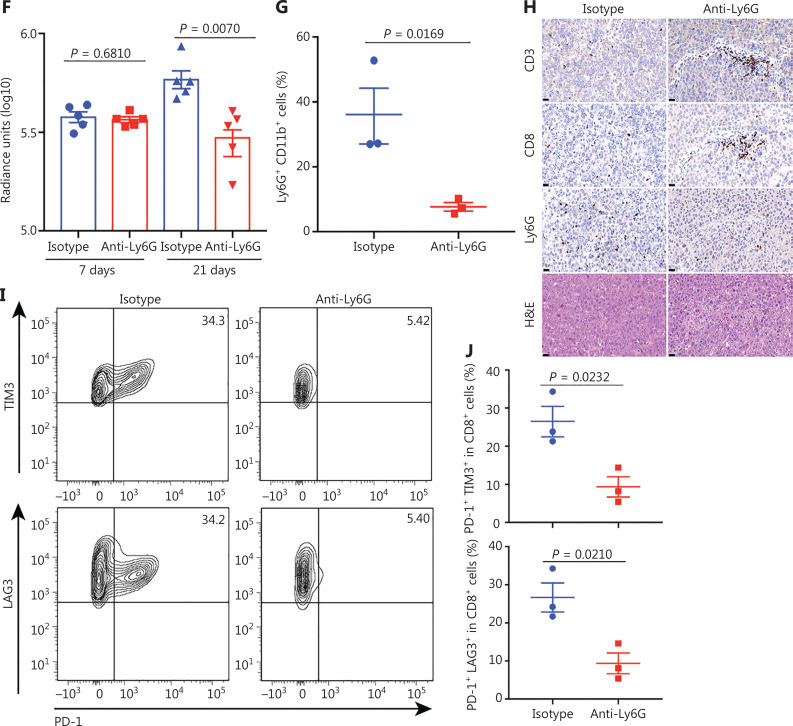
Tumor associated neutrophils promote CD8^+^ T cell exhaustion, thereby facilitating mouse liver cancer growth. (A) Schematic of the experimental approach. (B) Representative photographs of pTMK/Trp53^−/−^ mice treated with isotype control or anti-Ly6G antibodies, showing microscopic tumors. Ruler in the photo shows a minimum unit of mm. (C) The tumor burden was quantified by the ratio of liver weight to body weight. (D) Schematic of the therapeutic approach. (E) Bioluminescence signals showing tumor size in 2 groups at 7 and 21 days after injection of vectors. (F) Quantification of luciferase signals at 7 and 21 days after injection of vectors. (G) FACS analysis of neutrophil populations in the isotype control and anti-Ly6G groups. (H) H&E and IHC of CD3, CD8, and Ly6G in tumors or margin regions in mice treated with isotype control or anti-Ly6G antibodies. Scale bar: 50 μm. (I) FACS analysis showing dual positive PD-1^+^ TIM3^+^ CD8^+^ or PD-1^+^ LAG3^+^ CD8^+^ T cell proportions in 2 groups. (J) Dot plot showing statistical analysis of the FACS results.

Diverse functions of neutrophils have been observed during tumor development^13–19^. In our recent study, we have found that neutrophil subsets are largely conserved between humans and mice^24^. These data laid a basis for neutrophil-targeting therapy in pTMK mouse models. To determine the roles of neutrophils in our ICC/CHC liver cancer model, we intraperitoneally administered isotype (IgG2a) control or anti-Ly6G antibodies to mice to continually deplete neutrophils before pTMK plasmid injection (**Figure 3A**). The depletion of neutrophils significantly decreased the ratio of liver weight to body weight, and tumor development (**Figure 3B and 3C**).

To test the therapeutic potential of anti-Ly6G treatment, we fused luciferase to *Myc* *via* T2A in the transposon pTMK vector (pTMK-Luci), and monitored early tumorigenesis (**Figure 3D**). As shown in **Figure 3E**, we observed a bioluminescence signal by IVIS after 7 days of HDTVi, thus suggesting the initiation of tumor development. Mice with similar luciferase signals were randomly divided into 2 groups receiving treatment with anti-Ly6G antibodies or isotype controls. After 21 days of treatment, the bioluminescence signals of the tumors in mice treated with anti-Ly6G antibodies were significantly lower than those of the mice treated with the isotype control (**Figure 3E and 3F**).

To further explore the mechanism through which neutrophils support liver tumor development, we examined the TIME. Along with the depletion of neutrophils by anti-Ly6G antibody treatment, CD8^+^ T cells were markedly infiltrated into the tumors (**Figure 3G and 3H**). Moreover, the depletion of neutrophils decreased the number of PD-1^+^ TIM3^+^ CD8^+^ and PD-1^+^ LAG3^+^ CD8^+^ T cells (**Figure 3I and 3J**). Together, these results suggested that neutrophils inhibit CD8^+^ T cell tumor infiltration, promote their exhaustion, and thus support tumor development.

### TANs suppress CD8^+^ T cell activity

**Figure 4:**
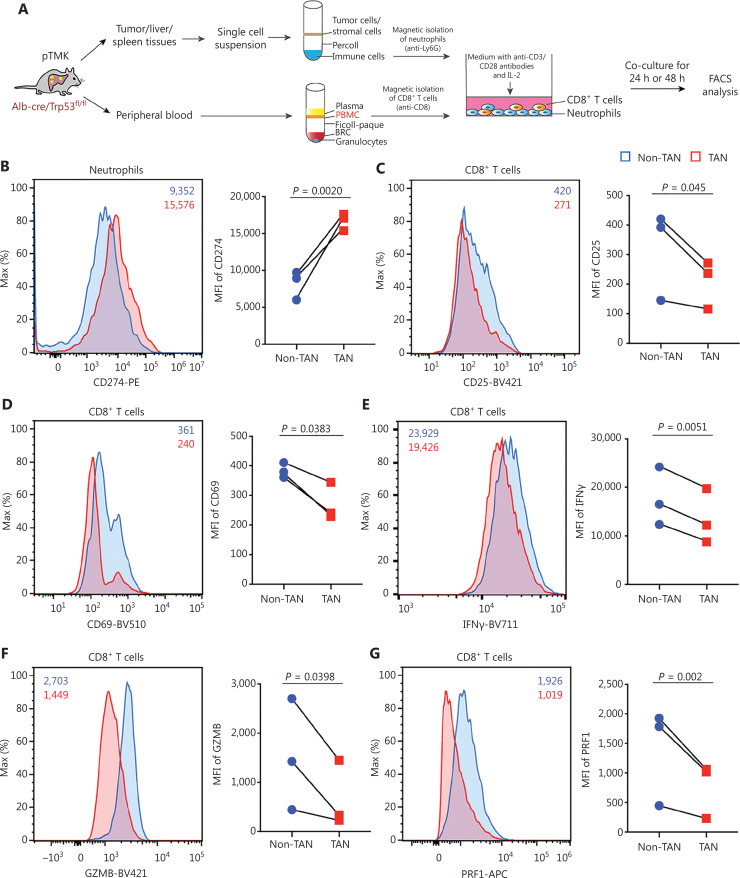
pTMK/Trp53^−/−^ tumor-associated neutrophils suppress CD8^+^ T cell activity. (A) Schematic of the experimental workflow. (B) FACS analyses and dot plot showing elevated PD-L1 expression in TANs from tumor tissues from pTMK/Trp53^−/−^ mice. (C-D) FACS analyses and dot plot showing decreased CD25 and CD69 expression in autologous CD8^+^ T cells co-cultured with TANs. (E-G) FACS analyses and dot plot showing decreased expression of cytotoxic markers (IFNγ, GZMB, and PRF1) in autologous CD8^+^ T cells co-cultured with TANs.

To further validate the effects of TANs on CD8^+^ T cells, we isolated TANs, adjacent liver neutrophils, PBNs, and SNs from pTMK/Trp53^−/−^ mice and cocultured them with autologous CD8^+^ T cells (**Figure 4A**). The expression of PD-L1 in the TANs was significantly higher than that in the non-TANs (ALNs, PBNs, or SNs) (**Figure 4B**). Moreover, the expression of the activation markers CD25 and CD69 on CD8^+^ T cells decreased when the cells were cocultured with TANs but not non-TANs (**Figure 4C and 4D**), thus suggesting that liver cancer TANs directly inhibited CD8^+^ T cell activation. Furthermore, the decrease in the expression of IFNγ, GZMB, and PRF1 in T cells cocultured with TANs suggested that TANs notably suppressed CD8^+^ T cell cytotoxicity (**Figure 4E-4G**). Together, these results suggested that TANs from pTMK/Trp53^−/−^ mice directly suppress the activation and cytotoxicity of CD8^+^ T cells *ex vivo*.

### CD8^+^ T and NK cells are essential for the therapeutic efficacy of neutrophil depletion

**Figure 5:**
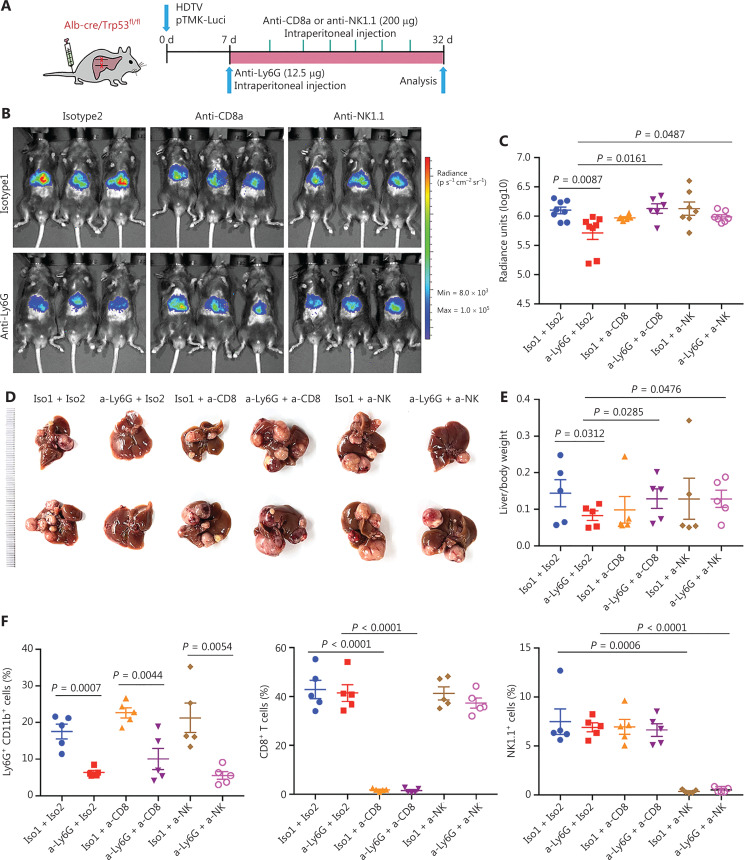
CD8^+^ T and NK cells are essential for the therapeutic efficacy of anti-Ly6G blockade. (A) Schematic of the experimental approach. (B) Bioluminescence signals showing tumor sizes in all treatment groups. (C) Quantification of luciferase signals with the treatment of corresponding antibodies. (D) Representative image of microscopic tumors from the indicated groups. Ruler in the photo shows a minimum unit of mm. (E) Quantification of tumor burden according to the ratio of liver weight to body weight. (F) FACS analysis showing the depletion efficiency of neutrophils, CD8^+^ T and NK cells with corresponding antibodies.

To further validate the roles of CD8^+^ T cells in response to anti-Ly6G therapeutic efficacy, we treated pTMK/Trp53^−/−^ mice with anti-CD8a antibodies to deplete cytotoxic CD8^+^ T cells in combination with anti-Ly6G antibody treatment (**Figure 5A**). Depletion of neutrophils slowed tumor growth; however, when CD8^+^ T cells were depleted, the antitumor efficacy of anti-Ly6G was abolished (**Figure 5B-5E**). The depletion efficacy of related immune cells by the antibodies was confirmed (**Figure 5F**). These results suggested that the antitumor effect of the anti-Ly6G antibody was dependent on CD8^+^ T cells.

Interestingly, the depletion of NK cells also diminished the therapeutic effects of anti-Ly6G (**Figure 5A-5F**). Li^34^ has reported that lung-infiltrating neutrophils suppress CD8^+^ T cells and NK cells *via* inducible nitric oxide synthase or reactive oxygen species in premetastatic breast cancer. These findings suggest that TANs may play a crucial role in NK cell cytotoxicity and support our hypothesis that neutrophil-targeting therapy for liver tumors is dependent on NK cells.

Together, the results confirmed that TANs in the pTMK/Trp53^−/−^ ICC/CHC tumor microenvironment suppress antitumor immune cells and consequently support liver cancer development.

### TANs have synergistic effects with ICB in the TIME of liver cancer

**Figure 6:**
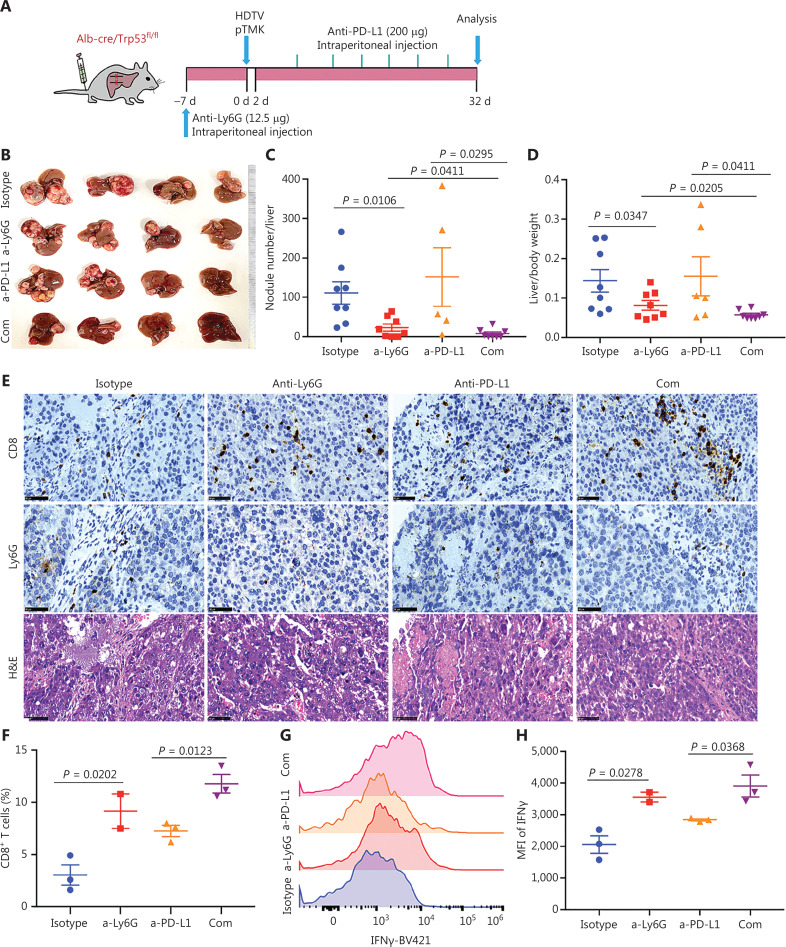
Tumor associated neutrophils are the key negative regulators of ICB in mice with liver cancer. (A) Schematic of the experimental workflow. (B) Representative image of microscopic tumors from mice treated with single agents alone or the indicated combinations. Ruler in the photo shows a minimum unit of mm. (C-D) Tumor burden, quantified by nodule numbers per liver and the ratio of liver weight to body weight. (E) H&E and IHC of CD8, Ly6G staining in tumor areas or margins in mice after different treatments. Scale bars: 20 μm. (F) FACS analysis, showing the CD8^+^ T cell populations in 4 groups. (G-H) FACS analysis, showing the expression of IFNγ in CD8^+^ T cells from all groups.

To determine whether the depletion of neutrophils might further increase the efficacy of ICB, we treated pTMK/Trp53^−/−^ mice with anti-Ly6G antibodies in combination with anti-PD-L1 antibody treatment (**Figure 6A**). Similar to anti-PD-1 monotherapy, treatment with anti-PD-L1 antibody alone did not inhibit tumor growth (**Figure 6B-6D**). However, the combination of anti-PD-L1 antibody treatment with neutrophil depletion markedly decreased tumor growth in terms of both the number of tumor nodules and overall tumor weight (**Figure 6B-6D**). Moreover, tumors treated with anti-Ly6G plus anti-PD-L1 antibodies showed the greatest accumulation of tumor-infiltrating CD8^+^ T cells inside tumors (**Figure 6E**). More importantly, cytotoxic CD8^+^ T cells significantly increased in response to the combination of neutrophil depletion and PD-L1 blockade (**Figure 6F and 6H**). Together, these results indicated that the depletion of TANs may synergistically improve ICB therapy in liver cancer.

## Discussion

The genomic heterogeneity of liver cancer implies that genetic alterations drive diverse liver cancers^5–9,35^. The tumor suppressor *Tp53* is one of the most frequently altered genes in patients with liver cancer, exhibiting deletion or mutation in almost half of the patients^6,9^. Amplification of the oncogene *Myc* is also common in liver cancer^6,9^. Oncogenic *Kras* mutations, such as *Kras*^G12D^, occur in 19% of patients with liver cancer^9^. *Myc* amplification and *Kras* mutation in patients with liver cancer are highly co-occurring^6,9^. Here, we used a transposon-based *Myc* and *Kras*^G12D^ overexpression system, and HDTV injection, to integrate oncogenes into the liver genome in conditional Trp53 null/null mice. All pTMK/Trp53^−/−^ mice developed ICC and CHC within 40 days, and had a median survival time of 35–50 days. This model, which displayed rapid tumor formation, allowed us to study antitumor immunity in certain types of liver cancer.

Genomic heterogeneity in cancer cells may further determine the immune microenvironment^25–29^. Our recent study has defined 5 distinct TIME subtypes in human primary liver cancer^24^. The Trp53 gene is altered in all 5 subtypes, and *Kras* mutations are present in suppressive myeloid and stromal cells, whereas Ctnnb1 mutations, another driver gene found in 16% of liver cancer cases, are associated primarily with immune exclusion and immune intermediates^9,24^.

Patients with ICC carrying *Kras* mutations are potentially resistant to anti-PD-1 therapies^7,8,36^. A phase II trial study of patients with advanced biliary tract cancer, which comprises ICC, extrahepatic cholangiocarcinoma, and gallbladder cancer, has shown that the objective response rate to pembrolizumab is poor, at 5.8%^37^. In agreement with these clinical observations, pTMK/Trp53^−/−^ tumors with ICC and CHC phenotypes did not respond to anti-PD-1 blockade. Although we observed a mild increase in CD8^+^ T cell infiltration in tumors of pTMK/Trp53^−/−^ mice after anti-PD-1 antibody treatment, CD8^+^ T cells might be trapped by TANs, TAMs, and CAFs in the suppressive TIME of liver cancer. Thus, this novel mouse ICC/CHC model may serve as a unique and valuable tool to study the mechanisms of the immune landscape in liver cancer.

The myeloid suppressive TIME may be induced by *Kras*-mediated signaling in cancer cells^7,8,36,38^. For example, oncogenic *Kras* stimulates the production of GM-CSF in human cancer cells, thereby recruiting myeloid cells that suppress immune surveillance^38^. Downstream products of oncogenic *Kras*-mediated signaling, such as IL1β, IL-6, VEGF, and GM-CSF, induce the recruitment of CD11b^+^Gr1^+^ neutrophils in a variety of mouse tumors, thus leading to T cell suppression^38,39^. In addition, the activation of MAPK, PI3K-Akt, YAP-TAZ, and JAK-STAT3 signaling pathways by *Kras* in pancreatic cancer cells results in the upregulation of various suppressive cytokines and chemokines, such as IL-4, IL-6, IL-13, CSF1, and MCP-1^40,41^. These molecules subsequently attract and enhance the survival and infiltration of immune cells that possess suppressive properties, including CAFs, TANs, TAMs, suppressive DCs, and Tregs, which in turn reinforce the immunosuppressive environment within the tumors^41^. In contrast, pTMC/Trp53^−/−^ mice in which *Myc* and Ctnnb1 oncogenes are integrated exhibit liver cancer with HCC phenotypes^24^. Few cytokines have been detected in CTNNB1-mutant tumors; however, chemokines such as CXCL1, CCL20, and CCL5 have been found to be significantly diminished in both murine and human CTNNB1-mutant tumors^31–33^. Expression of CCL5 in CTNNB1-driven tumors increases the DC and CD8^+^ T cell populations, thereby restoring immune surveillance^31^.

**Figure 7:**
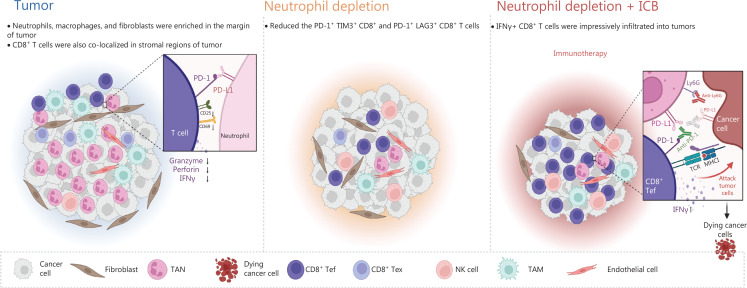
Schematic of the mechanism through which neutrophils, as key regulators of tumor immunity, restrict immune checkpoint blockade in liver cancer. The liver tumors with ICC and CHC displayed moderate responses to anti-Ly6G treatment but not PD-1 blockade. Depletion of neutrophils increased the infiltration of CD8^+^ T cells and decreased the exhausted T cells in the tumor microenvironment. Moreover, a combination of anti-Ly6G with anti-PD-L1 enhanced the infiltration of cytotoxic CD8^+^ T cells, thus resulting in a significantly greater decrease in tumor burden. CD8^+^ Tef: CD8^+^ effector T cell; CD8^+^ Tex: CD8^+^ exhausted T cell.

TANs are key players in the formation of the immune suppressive microenvironment^24^. The interactions among tumor cells, TANs, and CD8^+^ T cells in tumor progression and ICB resistance is a complex and active area of research. Evidence has suggested that TANs impede the activity of CD8^+^ T cells and induce immune suppression, which facilitates tumor growth. Whereas, CD8^+^ T cells are essential for eliminating tumors and enhancing the efficacy of ICB therapy. Recent studies have delved into the mechanisms behind the relationship between TANs and CD8^+^ T cells. For example, our recent study published in Nature in 2022 demonstrated that CCL4^+^ TAN promotes tumor growth by recruiting tumor-associated macrophages, whereas PD-L1^+^ TANs promote tumor growth by inhibiting the killing function of CD8^+^ T cells^24^. Another study published in Advanced Science in 2023 has indicated that tumor-intrinsic Setd2 deficiency enhances recruitment and reprogramming of neutrophils in pancreatic tumors, thereby escaping from immune surveillance *via* inhibiting the cytotoxicity of CD8^+^ T cells^42^. However, more research is needed to fully understand the complex interactions between these cells and the signaling pathways involved in tumor progression and ICB resistance. Future studies could explore the potential of targeting TANs to enhance CD8^+^ T cell activity and improve the response to ICB therapy. Our results indicated that PD-L1-expressing TANs substantially suppressed CD8^+^ T cell activation and cytotoxicity. In pTMK/Trp53−/− mice, reducing neutrophils effectively reduced tumor burden. This reduction allowed for an increase in the infiltration of CD8^+^ T cells with exhaustion phenotypes. Removing CD8^+^ T cells weakened the inhibitory effects of neutrophil depletion, indicating that CD8^+^ T cells played a role in eliminating liver cancer cells. The depletion of NK cells also impaired the inhibitory effect of neutrophil depletion, highlighting the importance role of NK cells in this treatment. Additionally, neutrophil depletion promoted TIME reprogramming and worked synergistically with the PD-1/PD-L1 immune checkpoint to produce the most significant decrease in tumor burden compared to the single treatment of neutrophil depletion or the administration of PD-1. Therefore, targeting immunosuppressive neutrophils could be a promising treatment approach for liver cancer (**Figure 7**).

Together, these findings suggest a possible mechanism by which tumors bearing *Kras* mutation recruit neutrophils and convert them into TANs, leading to increased immunosuppression *via* the formation of an immune barrier that inhibits the entry of cytotoxic CD8^+^ T cells, ultimately causing immune resistance. The combination of anti-PD-L1 and anti-Ly6G therapies disrupted this barrier, thereby facilitating the infiltration of more cytotoxic CD8^+^ T cells into the tumor tissue to target tumor cells. As a result, the synergistic effect of neutrophil depletion and ICB in our GEMM (Genetically Engineered Mouse Models) mouse tumor therapy has significant implications for prospective clinical trials in the future.

## Conclusions

In summary, our study elucidated that the myeloid suppressive microenvironment of liver tumors leads to resistance to ICB. To explore the mechanism through which TANs affect the antitumor immune response, we performed IHC staining to examine the CD8^+^ T cell distribution. Ly6G depletion led to tumor infiltration of CD8^+^ T cells from tumor marginal areas. We hypothesized that Ly6G depletion might affect the interaction of TANs and CD8^+^ T cells rather than CD8^+^ T cell priming by chemokine secretion. Subsequently, FACS assays demonstrated that TANs expressed PD-L1, which in turn suppressed CD8^+^ T cell activation and cytotoxicity (**Figure 4**). Hence, TANs may form an immunosuppressive TIME that inhibits CD8^+^ T cell activity, and neutrophil depletion decreases tumor burden. Neutrophil depletion may reprogram TIME suppression and have synergistic effects with ICB therapy. Together, our findings suggested that targeting these immunosuppressive cells may be a promising treatment strategy for liver cancer.
