# Supplementary material for: Neutrophils as key regulators of tumor immunity that restrict immune checkpoint blockade in liver cancer
Source: Cancer Biol Med. 2023 May 13;20(6):421–37. doi: 10.20892/j.issn.2095-3941.2023.0019 (PMC10291984; doi:10.20892/j.issn.2095-3941.2023.0019)
Supplement: Supplementary file 1 [file cbm-20-421-s001.pdf]

Supplementary materials

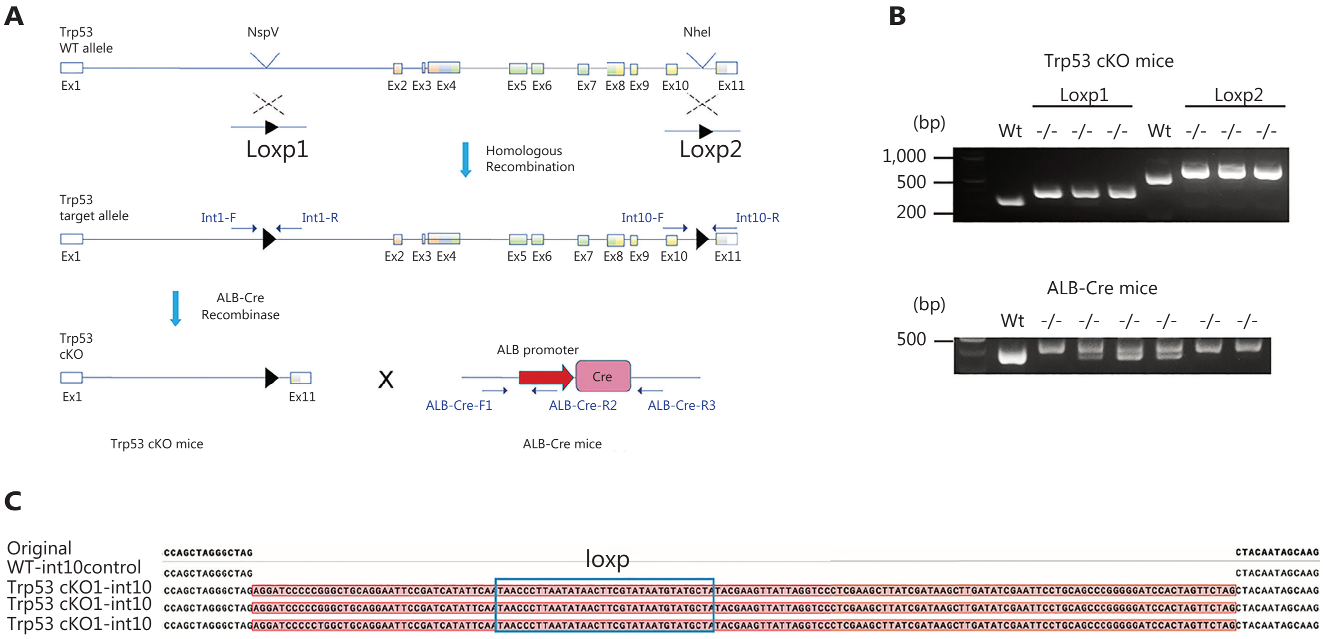

Figure S1 The basic information of Trp53 cKO (conditional knockout) mice. (A) Schematic depicting the endogenous Trp53 locus, *Tp53* flox mice have loxP sites flanking exons 2-10 of the transformation related protein 53 gene. Exposure to Cre recombinase removes the floxed sequence - creating a null allele. When bred to mice with a Cre recombinase gene under the control of a ALB promoter, expression is deleted in the tissue of interest. (B) PCR for genotyping the Trp53 flox and the ALB-CRE alleles. For Trp53 flox genotyping-Mutant = 390 bp, Heterozygote = 270 bp and 390 bp, Wild type = 270 bp. For ALB-CRE genotyping. Mutant = ~390 bp Heterozygote = ~390 bp and 351 bp, Wild type = 351 bp. (C) The locus and sequence of loxP site in Trp53 cKO mice.

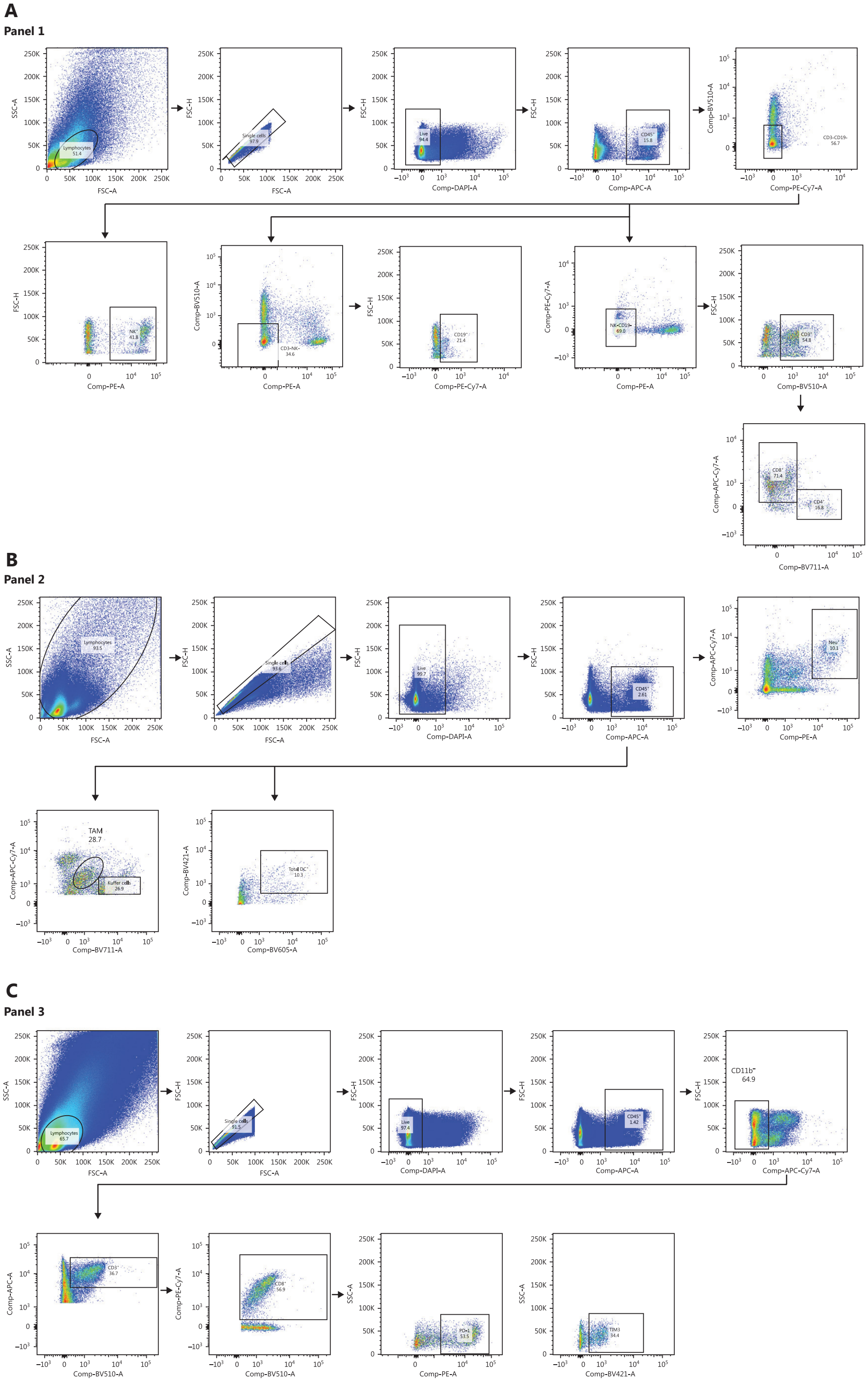

Figure S2 (A) Gating strategy and illustrative plots of lymphoid cells of a representative sample. (B) Gating strategy and illustrative plots of myeloid cells of a representative sample. (C) Gating strategy and illustrative plots of CD8⁺ T cells status of a representative sample.
